# Evaluation of a community health worker intervention and the World Health Organization’s Option B versus Option A to improve antenatal care and PMTCT outcomes in Dar es Salaam, Tanzania: study protocol for a cluster-randomized controlled health systems implementation trial

**DOI:** 10.1186/1745-6215-15-359

**Published:** 2014-09-15

**Authors:** David Sando, Pascal Geldsetzer, Lucy Magesa, Irene Andrew Lema, Lameck Machumi, Mary Mwanyika-Sando, Nan Li, Donna Spiegelman, Ester Mungure, Hellen Siril, Phares Mujinja, Helga Naburi, Guerino Chalamilla, Charles Kilewo, Anna Mia Ekström, Wafaie W Fawzi, Till W Bärnighausen

**Affiliations:** Department of Global Health and Population, Harvard School of Public Health, Huntington Avenue, Boston, Massachusetts 02115 USA; Management and Development for Health, Mwai Kibaki Road, Dar es Salaam, Tanzania; Departments of Epidemiology and Biostatistics, Harvard School of Public Health, Huntington Avenue, Boston, Massachusetts 02115 USA; School of Public Health, Muhimbili University of Health and Allied Sciences, United Nations Road, Dar es Salaam, Tanzania; School of Medicine, Muhimbili University of Health and Allied Sciences, United Nations Road, Dar es Salaam, Tanzania; Department of Public Health Sciences, Tomtebodavägen, Karolinska Institutet, Solna, SE-171 77 Stockholm Sweden; Department of Infectious Diseases, Karolinska University Hospital, Karolinskavägen, Solna, SE-171 76 Stockholm Sweden; Wellcome Trust Africa Centre for Health and Population Studies, A2074 Road, Mtubatuba, KwaZulu-Natal 3935 South Africa

**Keywords:** Study protocol, HIV, Antenatal care, Prevention of mother-to-child transmission, Community health workers, Uptake, Retention

## Abstract

**Background:**

Mother-to-child transmission of HIV remains an important public health problem in sub-Saharan Africa. As HIV testing and linkage to PMTCT occurs in antenatal care (ANC), major challenges for any PMTCT option in developing countries, including Tanzania, are delays in the first ANC visit and a low overall number of visits. Community health workers (CHWs) have been effective in various settings in increasing the uptake of clinical services and improving treatment retention and adherence. At the beginning of this trial in January 2013, the World Health Organization recommended either of two medication regimens, Option A or B, for prevention of mother-to-child transmission of HIV (PMTCT). It is still largely unclear which option is more effective when implemented in a public healthcare system. This study aims to determine the effectiveness, cost-effectiveness, acceptability, and feasibility of: (1) a community health worker (CWH) intervention and (2) PMTCT Option B in improving ANC and PMTCT outcomes.

**Methods/Design:**

This study is a cluster-randomized controlled health systems implementation trial with a two-by-two factorial design. All 60 administrative wards in the Kinondoni and Ilala districts in Dar es Salaam were first randomly allocated to either receiving the CHW intervention or not, and then to receiving either Option B or A. Under the standard of care, facility-based health workers follow up on patients who have missed scheduled appointments for PMTCT, first through a telephone call and then with a home visit. In the wards receiving the CHW intervention, the CHWs: (1) identify pregnant women through home visits and refer them to antenatal care; (2) provide education to pregnant women on antenatal care, PMTCT, birth, and postnatal care; (3) routinely follow up on all pregnant women to ascertain whether they have attended ANC; and (4) follow up on women who have missed ANC or PMTCT appointments.

**Trial registration:**

ClinicalTrials.gov:EJF22802. Registration date: 14 May 2013.

**Electronic supplementary material:**

The online version of this article (doi:10.1186/1745-6215-15-359) contains supplementary material, which is available to authorized users.

## Background

### Mother-to-child transmission of HIV

The Joint United Nations’ Programme on HIV/AIDS (UNAIDS) estimates that 260,000 children globally were newly infected with HIV in the year 2012[1], the vast majority through vertical transmission of the virus from mother to child. Thus, even though the number of HIV infections in children decreased by an estimated 52% between 2001 and 2012, vertical transmission remains a significant problem, particularly in sub-Saharan Africa (SSA)[1]. Most of these infections are preventable. The World Health Organization (WHO) estimates that in the absence of any intervention the mother-to-child transmission (MTCT) rate is 15 to 45%[2]. Several studies in low-and middle-income countries have shown that by using maternal triple antiretroviral (ARV) regimens it is possible to reduce the six-month MTCT rate to less than 5%, even in low-resource settings (Table 1). The MTCT rate at six months is often used as an outcome in studies on prevention of MTCT (PMTCT). However, if breastfeeding continues beyond six months of age, HIV can still be transmitted.

**Table 1:** **Studies conducted in low-and middle-income countries, which achieved a six-month mother-to-child HIV transmission rate of ≤5%**

Country	Year	Participants	Maternal regimen	Infant regimen	Transmission rate at 6 months (95% confidence interval)
Botswana[3]	2006-2008	730 breastfeeding women^1^	Triple ARVs from 28-34 weeks until cessation of breastfeeding^2^	sd-NVP + AZT for 4 weeks	1.1% (0.5%-2.2%)
Rwanda[4]	2005-2007	Mothers of 227 breastfed and 305 formula-fed infants	Formula-feeding: triple ARVs from 28 weeks until birth. Breastfeeding: triple ARVs until cessation of breastfeeding^2^	sd-NVP + AZT for 7 days	Breastfed: 1.8%^3^ (0.7%-4.8%)
					Formula-fed: 1.0%^3^ (0.3%–3.0%)
Mozambique[5]	2005-2007	341 breastfeeding mother-infant pairs	Triple ARVs from 15 weeks until cessation of breastfeeding^2^	sd-NVP + AZT for 7 days	2.1%
Mozambique, Malawi, and Tanzania[6]	2004-2006	809 formula-feeding women	Triple ARVs from 25 weeks until cessation of breastfeeding^2^	sd-NVP + AZT for 7 days	2.7%
Burkina Faso, Kenya, South Africa[7]	2005-2008	Mothers of 805 infants^4,5^	Group 1: triple ARVs from 28-36 weeks until cessation of breastfeeding^2,6^	sd-NVP + AZT for 7 days	Group 1: 4.9% (3.1%-7.6%)
			Group 2: AZT during pregnancy + sd-NVP and AZT at onset of labor^7,8^		Group 2: 8.4% (6.0%-11.6%)
Kenya[8]	2003-2009	Mothers of 487 breastfed infants	Triple ARVs from 34-36 weeks until cessation of breastfeeding^2^	sd-NVP	5.0% (3.4%-7.4%)
Tanzania[9]	2004-2006	Mothers of 441 breastfed infants	Triple ARVs from 34 weeks until cessation of breastfeeding^2^	AZT + 3TC for 7 days	5.0% (3.2%-7.0%)

3TC, lamivudine; ARV, antiretroviral drugs; AZT, zidovudine; NVP, nevirapine; sd-NVP, single dose of nevirapine at birth.

^1^Of these 730 women, 560 had a CD4-count ≥200 cells/mm^3^ and 170 a CD4-count <200 cells/mm^3^ or an AIDS-defining illness.

^2^Women were advised to have concluded complete cessation of breastfeeding by 6 to 7 months postpartum, depending on the study.

^3^These are transmission rates at 9 months (not 6 months).

^4^78% of infants born to women in each arm of the study were ever breastfed.

^5^This is the number of participants randomized at the beginning of the study.

^6^349 infants’ mothers were provided with this regimen.

^7^339 infants’ mothers were provided with this regimen.

^8^After a protocol amendment in 2006, women in the AZT arm also received AZT + 3TC 7 days postpartum.

### The obstacles of late and low antenatal care uptake and retention

WHO recommends the integration of PMTCT services with maternal and child health services[10]. In integrated PMTCT services, antenatal care (ANC) is the key entry point for PMTCT services (for example, through HIV testing). Therefore, in many low- and middle-income countries, one of the major challenges to achieving the maximum benefits from PMTCT services is late and low ANC attendance. The first ANC visit is often delayed and women attend a low overall number of ANC visits[11, 12]. WHO recommends that pregnant women have their first ANC visit in the first trimester of pregnancy and attend a total of at least four visits[13]. In Tanzania, an estimated 98% of women attended at least one ANC visit for their most recent live birth[14]. However, only 43% made four ANC visits or more, and only 15% of women attended ANC during the first trimester of pregnancy (Additional file1). Late and inconsistent attendance of ANC means that any pre-existing health problems (such as sexually transmitted infections and anemia) as well as problems arising during pregnancy (such as urinary tract infections, malaria, gestational diabetes, and pre-eclampsia) are more likely to be detected late, thereby significantly increasing the risks of adverse outcomes from these conditions to the health of the mother and the newborn child[15]. Moreover, depending on the delay in the first ANC visit, HIV-infected women may not be initiated on ARVs at all or may receive ARVs late in pregnancy, decreasing the effectiveness and efficacy of PMTCT[7].

### Community health worker interventions to improve ANC and PMTCT outcomes

This cluster randomized health systems implementation study, called the *Familia Salama* (Kiswahili for ‘safe family’) trial, will test the effectiveness, cost-effectiveness, feasibility, and acceptability of a community health worker (CHW) intervention in improving ANC and PMTCT outcomes. While there is considerable evidence to show that CHW interventions can contribute to child health improvements[16, 17], the evidence for the effect of CHW interventions on reproductive health services (including ANC and PMTCT) is scant[16, 18]. A systematic review by WHO on CHW interventions has found that for interventions with a maternal health component "almost all of the CHW-driven interventional studies showed (…) a[n] improvement in perinatal and postpartum service utilization indicators" (WHO 2010)[16], but it noted that few studies were conducted at large scale and none had employed an experimental design. In addition, it found a "remarkable dearth of information" on the cost-effectiveness of CHW programs[16]. Concerning PMTCT, the WHO review did not identify a single study of a CHW intervention to increase PMTCT uptake and/or retention[16]. Nonetheless, a more recent systematic review by UNAIDS has identified several examples of successful programs that use CHWs to improve PMTCT uptake and retention. However, none of these programs were evaluated in a randomized trial[19].

The *Familia Salama* trial will thus provide much needed evidence on the effectiveness of community health workers (CHWs) in increasing ANC and PMTCT uptake and retention. The results of this study will be particularly important as it is a large-scale randomized health systems implementation trial, which also assesses the acceptability, feasibility and cost-effectiveness of the CHW intervention.

### WHO’s PMTCT Option A and Option B

HIV can be transmitted from mother to child during pregnancy or childbirth through blood, or postnatally through breast milk. Until the early 2000s, PMTCT trials and WHO recommendations focused on preventing the transmission of HIV during pregnancy, birth, and the early postnatal transmission period. When WHO published updated PMTCT guidelines in 2000, randomized clinical trials had not tested PMTCT regimens with ARVs beyond seven days postpartum for the mother and six weeks postpartum for the infant[20]. Transmission through breast milk after the first few weeks of life, however, remained a serious challenge. Given the overwhelming benefits of breastfeeding, the lack of safe alternatives to human milk in resource-limited countries, and the documented risk of HIV transmission through breastfeeding, there was an urgent need to make breastfeeding by HIV-infected women safer by preventing postnatal transmission of the virus[21, 22].

In this context, research started to focus on how ARVs can be given to the mother or the infant in the postpartum period to reduce transmission rates while allowing the infant unrestricted access to breast milk. These studies showed promising results for prophylactic ARVs administered to infants[9, 22–26]. The scientific evidence from these studies, along with new evidence on safe feeding practices for HIV-exposed infants, early infant diagnosis, and revised maternal ARV eligibility, led to the updating of WHO’s PMTCT guidelines in 2010[10].

In its 2010 guidelines, WHO recommended the immediate initiation of lifelong triple ARV treatment for all HIV-infected pregnant women with a CD4 cell count of ≤350 cells/mm^3^, or who are in WHO clinical stage III or IV of HIV disease irrespective of CD4 cell count[10]. All infants born to pregnant women on lifelong triple ARV treatment should receive nevirapine (NVP) or zidovudine (AZT) for four to six weeks regardless of feeding mode. This is in contrast to the 2006 guidelines, which recommended lifelong treatment for HIV-infected pregnant women if the CD4 cell count was <200 cell/mm^3^, the woman was in clinical stage IV, or the woman was in clinical stage III with a CD4 cell count of ≤350 cells/mm[27].

For pregnant women who are not eligible for lifelong ARV treatment, the WHO 2010 guidelines recommend either of two drug regimens for PMTCT. The recommended maximum duration of breastfeeding, regardless of the PMTCT option used, is 12 months unless a nutritionally adequate and safe diet cannot be provided without breast milk[10].

Option A: AZT prophylaxis from as early as 14 weeks of gestation until delivery. If the woman has received AZT for less than four weeks, then NVP (single dose) + AZT + lamivudine (3TC) should be administered every 12 hours during labor and delivery, and AZT + 3TC for seven days postpartum. If the woman has received AZT for more than four weeks, another option is to continue AZT every 12 hours during labor and then stop ARV treatment. Breastfeeding infants should be given NVP daily until one week after stopping breastfeeding and non-breastfeeding infants should be given NVP or NVP (single dose) + AZT for four to six weeks after delivery.

Option B: one of the following triple ARV regimens from as early as 14 weeks of gestation until one week after stopping breastfeeding: (1) AZT + 3TC + lopinavir/ritonavir (LPV/r), (2) AZT + 3TC + abacavir, (3) AZT + 3TC + efavirenz (EFV), (4) tenofovir disoproxil fumarate (TDF) + 3TC (or emtricitabine) + EFV. All infants regardless of feeding mode receive NVP or AZT for four to six weeks.

Both the WHO 2010 and 2013 guidelines found that the available evidence does not show whether one of the two PMTCT options described above is more efficacious than the other[10, 28]. The WHO 2010 guidelines (the most current guidelines available at the beginning of the trial period) recommended that policymakers choose the option more suited to their cultural context, health system, and economic constraints[10]. However, it is still unclear which option will be more effective, cost-effective, feasible, and acceptable when implemented in real-life public-sector health systems in SSA. For example, regarding Option B, questions concerning potential adherence fatigue during the breastfeeding period and harm to mothers and infants from prolonged exposure to ARVs still remain largely unanswered[29]. It is not known how such potential disadvantages compare to the operational drawbacks of Option A, such as the need for CD4 counts to determine the type of regimen to be initiated, the needed changes from antepartum to intrapartum and postpartum regimens, and the requirement for extended NVP administration in breastfeeding infants[29, 30]. Evidence, particularly from large-scale, randomized health system trials, on these questions is needed for policymakers to design locally appropriate and optimal PMTCT policies.

### WHO’s PMTCT Option B+

PMTCT has been a dynamic field in the past and recent changes in WHO’s policy recommendations have been rapid and not without controversy[29]. This trial was designed when the latest policy guidance document on PMTCT that WHO had published were the 2010 PMTCT guidelines. In 2013, WHO published a new guideline on the use of ARV drugs for treating and preventing HIV infection. In these updated guidelines Option A is no longer recommended[28]. In addition, WHO added the so-called Option B+ as an alternative to Option B. Under Option B+, all HIV-infected pregnant women are started on ARVs irrespective of CD4 count and as soon as they have been diagnosed as HIV-infected.

Even though WHO no longer recommends Option A, it is still widely used across SSA since the implementation of new PMTCT guidelines lag behind the WHO recommendations in many countries, and this trial will provide useful evidence for PMTCT programs in many low- and middle-income countries. In its 2013 guidelines, WHO states that ‘research is needed to determine how to optimize acceptability, adherence and retention on antiretroviral therapy (ART) in pregnant and breastfeeding women’ (p. 104)[28]. Improving acceptability and retention in PMTCT care are central aims of the *Familia Salama* trial’s CHW intervention. In addition, the trial will provide useful information for those sub-Saharan African countries considering a transition from Option A to Option B or Option B+. In this context, it is important to note that in this trial and for practical purposes Option B is very similar to Option B+, because Option B and Option B+ do not differ during the course of pregnancy and the breastfeeding period. It is only once a woman has stopped breastfeeding that she would either stop (under Option B) or continue her ART for life (under Option B+). Since the entire length of this study is only 17 months, most women participating in this trial will have received the same treatment in the Option B arms of the trial that they would have received under the Option B+ policy. An important difference, nevertheless, lies in the woman’s expectation regarding the duration for which she should take ARVs, which may have an influence on adherence and retention. By working with, and collecting data from clinics and hospitals in wards, which were required to change their PMTCT regimen from Option A (the standard of care in Tanzania) to Option B for this trial, we will gain important insights on the implementation challenges of transitioning from Option A to Option B or B+ .

### PMTCT in Tanzania

In 2010, the MTCT rate in Tanzania was 25%[31], with 77% of HIV-infected pregnant women[1] and 74% of HIV-exposed infants[32] receiving ARVs for PMTCT (Additional file1).

The government of Tanzania began offering PMTCT services in 2000[33]. These services have now been integrated into Tanzania’s reproductive and child health services and should, therefore, be provided by the clinical staff in ANC, labor and delivery, and postnatal care facilities. However, not all ANC facilities offer ARVs, in which case women are referred to the ART treatment program. In June 2012, Tanzania adopted WHO’s 2010 PMTCT guidelines and implemented Option A as the standard of care. Apart from the treatment and prophylaxis of pregnant women and their newborns with ARVs, Tanzania’s PMTCT program includes voluntary testing of pregnant mothers with pre- and post-test counselling, infant feeding counselling, and family planning. In addition, the program includes the testing of HIV-exposed infants using polymerase chain reaction (PCR) at four to six weeks and six months after delivery, and a rapid HIV-test after complete cessation of breastfeeding, usually between 9 and 18 months after delivery[34].

Tanzania has decided to implement lifelong triple ARVs for all HIV-infected pregnant women (Option B+) in 2013 with a graduated rollout. Because of this decision the trial management team, in accordance with the Data Safety and Monitoring Board and the Tanzanian Ministry of Health and Social Welfare, decided to stop the trial in May 2014 rather than in January 2015.

### Objectives

The aim of this study is to determine the effectiveness, cost-effectiveness, acceptability, and feasibility of the following two interventions when implemented in the healthcare system in Dar es Salaam, Tanzania: (1) a CHW intervention to increase ANC and PMTCT uptake and retention, (2) WHO Option A versus B for PMTCT, and (3) the interaction between the CHW intervention and the two PMTCT options.

## Methods/Design

### Study duration

The trial is being carried out over a period of 17 months from January 2013 to May 2014.

### Study setting

The *Familia Salama* trial is being carried out in the Kinondoni and Ilala districts, two of the three administrative districts of the Dar es Salaam region of Tanzania. In 2012, 69% (2,995,660 out of 4,364,541) of Dar es Salaam’s population lived in these two districts[35]. Dar es Salaam is highly urbanized and is Tanzania’s most densely populated region with 3,133 people per square kilometer[35].

Similar to other regions in Tanzania, virtually all women in Dar es Salaam attend at least one ANC visit. However, the percentage of women who deliver at a health facility (90%), have knowledge of MTCT (71% of women knew that drugs can reduce the risk of MTCT) and received HIV-counselling during ANC (85%) is considerably higher in Dar es Salaam than in other regions of Tanzania[14, 36]. Dar es Salaam also has the second highest HIV-prevalence (9% among women and men aged between 15 and 49 years) of any region in Tanzania[14].

### Study design

**Figure 1:**
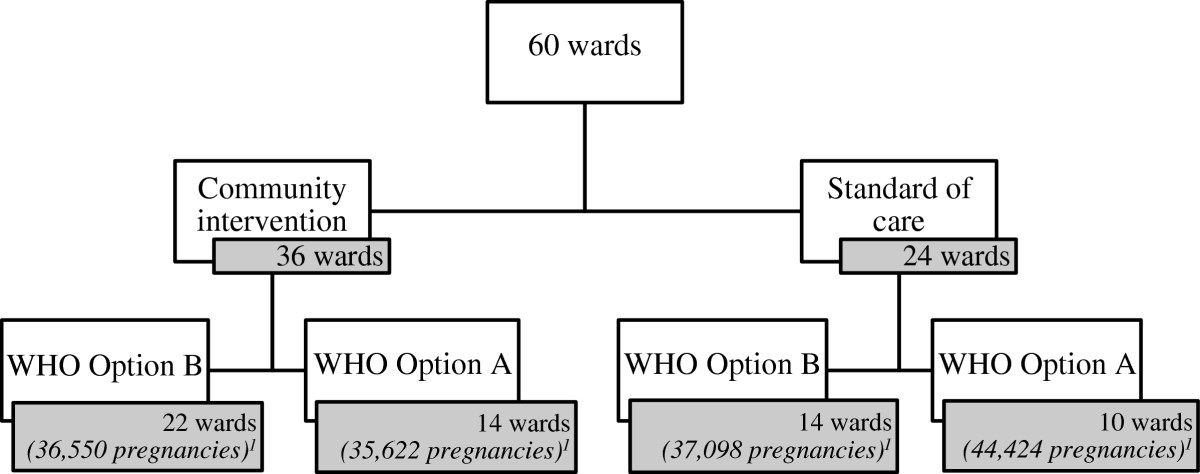
**The randomization scheme of the** ***Familia Salama*** **trial.**

The *Familia Salama* trial is a cluster-randomized controlled trial that uses a two-by-two factorial design (Figure 1). Together, the districts in which the trial takes place have 60 wards. These wards were randomly allocated to either the CHW intervention (36 wards) or the normal standard of care in Dar es Salaam (24 wards). The two arms were then randomized again to receiving either WHO Option A or Option B for PMTCT (Figure 1). The number of clusters in the arms varies to reflect the difference in the population sizes in each ward.

The study is carried out by Management and Development for Health (MDH, Tanzania). The Harvard School of Public Health provides technical assistance for the trial of the CHW intervention. MDH is a Tanzanian organization based in Dar es Salaam, which works in partnership with Tanzania’s Ministry of Health and Social Welfare. It provides technical and financial support to 50 HIV treatment sites, 17 tuberculosis clinics, and 180 PMTCT outlets across Dar es Salaam.

### The community health worker intervention

The CHW intervention employs two cadres of health workers that already exist in the public-sector health system in Tanzania. The first cadre is CHWs. This cadre is locally called home-based carers, or HBC, but we will continue to refer to them as community health workers, because the activities that these health workers carry out are similar to those carried out by CHWs in most other settings. The second cadre is nurses who are responsible for community outreach from clinical facilities and whose responsibility includes following up with HIV-infected pregnant women in the community when they miss a PMTCT appointment (see below). This cadre is locally referred to as community-based healthcare workers, or CBHC, but we will refer to them as community outreach nurses in this article, because this cadre is based in clinical facilities rather than in the community.

In wards randomized to the CHW intervention, the study received from the municipalities: (1) an additional 31 community outreach nurses to supplement the existing cadre of 21 community outreach nurses, with one to three nurses being assigned to each ward, and (2) a cadre of 215 CHWs, who work in the neighborhood in which their home is located. One to three CHWs were assigned to each street in the intervention wards depending on the size of the street.

### Responsibilities of the community health workers

While the CHWs exist as a cadre in the public-sector health system in Tanzania, they are currently not carrying out any specific activities to support ANC and PMTCT uptake and retention in the districts where the trial takes place. In the CHW intervention arms of the trial they are employed to fulfill the following functions: (1) identify pregnant women at the community level through home visits and refer them to ANC; (2) routinely revisit all identified pregnant women to check whether they have taken up ANC and to support first and future ANC uptake through information, education, and counselling; (3) visit pregnant women at home who have missed scheduled ANC or PMTCT appointments; (4) provide education to pregnant women and other household members on maternal health, general health issues (such as nutrition, immunization, and sanitation) and the importance of delivering at a healthcare facility, testing for HIV during ANC visits, PMTCT, breastfeeding, postnatal care, seeking early and appropriate healthcare in case of an illness during pregnancy. As is the standard in Dar es Salaam’s healthcare system, the CHWs receive a monthly stipend equivalent to $US35.

### Following up with women who have missed an ANC or PMTCT appointment

In control wards, only women who have missed a PMTCT appointment are tracked, while in wards randomized to the CHW intervention CHWs carry out home visits to pregnant women who have missed either a PMTCT or an ANC appointment. In addition, CHWs in the intervention wards routinely revisit all pregnant women to check whether they have attended ANC, until the women have attended ANC for the first time.

In the community intervention control areas, the community outreach nurses contact women once they are over seven days late for their ANC or PMTCT appointment to encourage them to attend. Contact is first attempted by phone, and subsequently by a home visit.

In the wards receiving the CHW intervention, the CHWs support the community outreach nurses in carrying out home visits to pregnant women. During the first ANC visit, the ANC nurse obtains the woman’s address and a written description of where and how her home can be found (a so-called map cue), as well as the woman’s telephone number. The community outreach nurses visit each ANC facility in their ward weekly to collect the addresses and telephone numbers of all women who are over seven days late for their ANC or PMTCT appointment. The community outreach nurses call these women to remind them of their appointment. If a community outreach nurse cannot reach a woman on the phone, s/he passes the information about the woman on to a CHW who will subsequently try to visit the woman. In addition, the community outreach nurses collect forms for referral to ANC from the CHWs and match them with the referral forms obtained from women at the ANC facilities. The community outreach nurses pass those referral forms for which there is no corresponding clinic visit (signifying that the woman was referred to ANC but did not attend) back to the CHW for follow-up at the woman’s home.

### Community sensitization activities

As part of the community health intervention, community sensitization activities are organized in intervention areas. The research team, along with project and municipal community health coordinators, provide information on maternal health and ANC to local leaders and encourages them to spread educational messages to their communities. The community leaders are the Ward Development Committee at the ward level and the Mtaa (Kiswahili for ‘neighborhood’) Executive Committee at the neighborhood level.

### The trial of WHO Option A versus Option B

WHO Option A is the current standard of care for PMTCT in Tanzania[34]. In the wards allocated to Option A, HIV-infected women who are not eligible for lifelong ARV therapy receive AZT from 14 weeks of gestation (or at the first PMTCT visit, if it takes place after the 14^th^ week of pregnancy) until delivery. This is followed by 12-hourly AZT + 3TC during labor and delivery and for seven days postpartum. If the woman has taken AZT for less than four weeks, an additional single dose of NVP is administered at the onset of labor. HIV-exposed breastfeeding infants are given NVP syrup once a day until one week after stopping breastfeeding while non-breastfeeding infants are given NVP syrup for six weeks after delivery. Tanzania’s PMTCT guidelines recommend that HIV-infected women breastfeed exclusively for the first six months and stop breastfeeding 12 months after birth unless an adequate diet cannot be provided to the infant without breast milk[34].

In the wards allocated to WHO Option B, HIV-infected women not eligible for lifelong ARVs receive AZT + 3TC + EFV from the 14^th^ week of pregnancy until one week after the complete cessation of breastfeeding. Regardless of the feeding option, all HIV-exposed infants in the Option B wards receive NVP syrup for four to six weeks after delivery.

Both PMTCT options are provided by clinical staff in ANC, labor and delivery, and postnatal care services. Some ANC facilities, however, do not provide ARVs, in which case the woman is referred to ART services.

HIV PCR testing for infants is the standard of care in Tanzania[34]. In all wards, PCR is performed at four to six weeks and six months after delivery and a HIV rapid diagnostic test (RDT) is administered after the complete cessation of breastfeeding, usually between 9 and 18 months of age. Infants’ CD4 count is measured every six months as per the national guidelines. HIV-infected pregnant women in all wards receive a daily double-dose of trimethoprim/sulfamethoxazole (Co-trimoxazole™), and exposed infants receive daily Co-trimoxazole™ syrup until their mother has stopped breastfeeding and they are confirmed to be HIV-uninfected.

### Patient and health worker satisfaction

Patient satisfaction is a determinant of treatment uptake, adherence and retention, as well as an important health systems outcome in its own right[37, 38]. Health worker satisfaction, on the other hand, is an important determinant of staff turnover[39], job-related motivation[40], and health worker migration[41]. It is plausible that the type of PMTCT option and the CHW intervention have effects on patient and health worker satisfaction. Option B requires more frequent visits to PMTCT facilities, which may reduce patient satisfaction and increase health workers’ workload and job dissatisfaction. At the same time, Option B has been widely hypothesized to be simpler to carry out than Option A, because it entails only one treatment for all HIV-infected pregnant and breastfeeding women[28]. This operational simplicity could conceivably improve patient and provider satisfaction. Similarly, the CHW intervention implies several changes that could affect patient and provider satisfaction. The home-based visits might lead to better or worse patient evaluation of the performance of the public-sector ANC and PMTCT systems. Leading and managing CHWs might improve job satisfaction among the community outreach nurses (since they now have greater spans of responsibility and have gained human resources to care for pregnant women in the community), or it might reduce job satisfaction (for example, if the community outreach nurses feel inadequately prepared for managing large numbers of CHWs).

In the *Familia Salama* study, we thus assess the causal effects of Option B (versus A) and the CHW intervention on patient and health worker satisfaction. To collect these data, we administer a questionnaire to patients and health workers in all four trial arms in the second year of the implementation of the interventions.

### Training

We have provided training in clinical PMTCT services to the facility-based health workers participating in this study to ensure that Option A and B are carried out as assigned through the trial. In addition, we have trained the CHWs and community outreach nurses in the delivery of the CHW intervention (Additional file2). The trainings were based on the Tanzanian National PMTCT Training Curriculum and the Tanzanian Community-Based Care Curriculum.

### Study endpoints

The primary endpoints of this study are: (1) the percentage of pregnant women making at least four antenatal clinic visits (as recommended by WHO); (2) the percentage of pregnant women delivering at a healthcare facility; (3) the percentage of HIV-infected women receiving PMTCT; (4) the percentage of HIV-exposed infants who received a confirmatory HIV test by six weeks after the cessation of breastfeeding; and (5) the percentage of infants born to HIV-infected mothers who have acquired HIV by 6 weeks after the complete cessation of breastfeeding.

The secondary endpoints of this study are: (1) the percentage of pregnant women who were tested for HIV during pregnancy or labor and delivery; (2) the number of weeks of gestation at which pregnant women attend ANC for the first time; (3) the percentage of HIV-infected pregnant women who completed PMTCT; and (4) the percentage of HIV-exposed infants who received PMTCT.

In addition to the designated primary and secondary endpoints, we will assess the causal impact of the interventions on a range of other endpoints, which capture the following outcomes: cost-effectiveness, social acceptability, patient satisfaction, and health worker satisfaction.

### Eligibility criteria

#### Inclusion criteria

All pregnant women who either attend ANC at any of the clinics included in this study or who are identified by the CHWs during the routine household visits as eligible for participation in this study.

#### Exclusion criteria

Any pregnant woman who is not able to provide verbal informed consent (for ANC attendees) or written informed consent (for PMTCT attendees) is ineligible to participate in the study.

### Randomization procedure

The unit of randomization is a ward. A ward in the Ilala and Kinondoni districts consists of two to ten streets and 6,742 to 106,946 inhabitants (mean: 49,928)[35]. The 60 wards in the Ilala and Kinondoni districts were divided into five strata by type of facility (military, high level, or standard level) and district (Ilala and Kinondoni). A ‘high level’ facility was defined as having the capacity to manage obstetric complications, including performing caesarian sections, while a ‘standard level’ facility does not have this capacity.

Based on prior empirical evidence, ANC and PMTCT outcomes differ significantly by facility type and district. Thus, the sample was stratified by these characteristics to ensure adequate representation of the different facilities in the final sample and to gain statistical efficiency. Within each stratum, the wards were sorted in descending order of expected number of pregnancies, based upon the number of pregnancies reported in August 2011. A block randomization was then applied. The first four wards in each stratum were randomly assigned to the four arms of the study. After each subsequent round of four assignments, the assignment probabilities were revised to increase the probability that the arms with lower numbers of pregnancies would be more likely to be chosen, thereby achieving the best balance across arms. This algorithm was repeated 500 times and the run giving the greatest balance in the expected number of pregnancies across the arms was selected.

While pregnant women can change the ANC facility they attend over the course of their pregnancy, clinicians were advised to maintain women on the PMTCT option on which they were started. Randomization into the CHW intervention is based on the ward in which the woman lives.

### Sample size

We expect that approximately 150,000 pregnancies would occur in the two districts during the study period, including 10,500 pregnancies among HIV-infected women. This figure is adjusted for the expected number of dropouts in the current standard of care, as it was estimated using data from the participating facilities’ ANC registration books from 2011. We calculated the minimum detectable difference between the control and the intervention arms for the primary endpoints, using methods for cluster-randomized trials[42] with unequal cluster sizes[40], with intra-cluster correlations between 0.001 and 0.030, as reported from similar cluster-randomized studies in low- and middle-income countries[43, 44]. Primary outcome rates were taken from Tanzanian national data and studies conducted in Botswana[45] and Zimbabwe[46]. The minimum effect sizes that can be detected with a Type I error of 0.05 are modest and well within the range of what could be expected from this study.

### Data collection

The majority of the study data is collected from clinical registers that are in routine use in the Tanzanian healthcare system (Additional file3). However, we have introduced five additional registers to record additional data for the purposes of this study. Data from all of these registers is entered by specialized data entry personnel into an electronic database written in Microsoft Access. A data quality manager checks for data entry error and data inconsistencies on an ongoing basis. In addition to the assessment of endpoints for the final study analysis, the data is further used to monitor study progress during weekly review meetings throughout the study period.

The study team runs quality checks on all backups received from the data entry personnel. These include the percentage of missing values for the variables in the register and standard error and plausibility checks. In addition, the number of women entered in each clinical register is compared to the monthly aggregate number reported by the district level authorities. Any inconsistencies in the data are discussed with individual data entry personnel and, if necessary, followed up on with visits to the healthcare facility and a review of the clinical register in question.

In addition, MDH has trained 30 nurses to mentor nurses at two to five other healthcare facilities. Clinical mentors visit their mentees’ facilities at least once a week. Mentorship activities focus on the accurate and complete filling out of clinical registers and ensuring the availability of HIV test kits and ARV drugs. The clinical mentorship program has been implemented in all wards in this trial.

### Economic evaluation

Based on our understanding of clinical activities, PMTCT Options A and B/B+ require different types of resources and different resource levels, but the real-life cost-effectiveness of implementing these various options in a setting like Dar-es-Salaam is not known. We will empirically measure resource utilization in all four intervention arms through a costing study. Health worker time, by cadre, for an average patient-case throughout the course of PMTCT will be estimated based on data from an empirical time-motion study. Study field workers with medical training will follow PMTCT nurses, health officers, and doctors during randomly selected clinic days to measure task times per PMTCT patient visit. These times will be used to estimate patient-case times, using the data available in the study database on the number and types of clinic visits per patient over the course of PMTCT. Other resource utilization, such as drugs and medical supplies, will be estimated based on standard clinical guidelines complemented by pharmaceutical and clinic records. Unit costs will be collected based on administrative data from the public-sector PMTCT program and procurement records, including health worker salaries, drug prices, prices of diagnostic tests, and healthcare facility rents.

### Data management

The study team trained a cadre of 20 data entry personnel in data entry and management. These data entry personnel work at the larger healthcare facilities in this study where desktop computers have been installed for data entry. When a clinical register is not in use, the data entry personnel enter the data from the register into a Microsoft Access database on the desktop computer. There is a separate password-protected Microsoft Access database for each register. In addition, some data entry staff form a mobile team that visits smaller healthcare facilities and enters data from the registers into a database on a laptop. To merge the data with the central database, the same data personnel take a password-protected backup of the clinic database on a laptop to the MDH headquarters at regular intervals (daily to monthly, depending on the size of the facility).

### Analysis plan

The data will be analyzed by intention-to-treat analysis at the participant level. Thus, participants will be assigned to the cluster in which they were resident at the start of the trial regardless of whether they move to other clusters or out of the trial areas during the study period. We will, however, still be able to determine the number of participants who crossed over between healthcare facilities or are lost to follow-up by linking women across clinic visits and clinical registers. To evaluate the effect of the interventions on the primary endpoints of this program, we will fit robust clustered log-binomial models[47]. The main primary endpoint of this trial is the percentage of infants born to HIV-infected mothers who have acquired HIV by 6 weeks after complete cessation of breastfeeding. Thus, the primary analysis will be run for this endpoint; other endpoints will be assessed in secondary analyses. The robust score test will be used to assess the statistical significance of intervention effects and the 95% confidence intervals will be based on the robust variance. In this way, the validity of inferences will not depend on the validity of assumptions about the correlation structure within or between wards, or on assumptions of normality or homoscedasticity of generalized regression residuals.

On average, randomization eliminates confounding; hence, the primary analysis will be unadjusted for potential confounding variables. However, because power can often be improved by covariate adjustment, secondary analyses will adjust for potential confounding for other measured individual-level risk factors such as age, as well as ward-level variables such as population size. To assess the statistical significance of effect modification, the likelihood ratio test will be used, comparing models with main effects only to models that include relevant cross-product terms. When significant effect modification is detected, point and interval estimates of intervention effects stratified by the effect modifier will be presented. Because effect modifiers are not hypothesized *a priori*, the study is not powered to detect effect modification and we will cautiously interpret any *post hoc* effect modification observed. All analysis will be conducted using the SAS statistical software package (SAS Institute, Cary, North Carolina).

### Interim analyses and stopping rules

The aggregate data (i.e., data not separated by assignment to intervention or control arms) for each endpoint will be analyzed continuously during this trial to monitor data quality. In addition, an independent Data Safety and Monitoring Board (DSMB) has been formed and meets biannually to review data quality and completeness, progress on the trial, adherence to the protocol, and the safety of participants. The board includes two Tanzanian and two United States-based scientists. In closed session, the study team’s statistician provides the DSMB with data on each endpoint disaggregated by assignment to intervention or control arms. No other research team members have access to this report.

There are no stopping rules for this trial because Option B is the PMTCT option recommended by WHO[28], and because it is unlikely that the CHW intervention could lead to adverse effects. However, safety endpoints are reviewed alongside the primary and secondary endpoints at each DSMB meeting. In addition, the DSMB was asked to review the trial protocol as new PMTCT guidelines, such as the WHO guidelines, are published. The DSMB, therefore, reviewed the study protocol when the new WHO 2013 guidelines were published and decided against amending the study design or discontinuing the trial.

### Ethical issues

#### Approvals

The National Institutes of Medical Research in Tanzania granted ethical approval for the study in June 2012. The institutional review board of the Harvard School of Public Health approved the analysis of the trial of the CHW intervention in January 2013; this study received continuing ethics approval in February 2014.

### Consent

Verbal consent is obtained in the CHW intervention at two time points: (1) by the CHWs before they enter a household for a counselling and information session; and (2) by the healthcare worker (a nurse or physician) at the first ANC visit. In the trial of WHO Option A versus Option B, the PMTCT health workers elicit written informed consent from HIV-infected pregnant women during their first PMTCT visit.

### Confidentiality

Participants’ confidentiality is protected several processes.

Firstly, for the information sessions provided during home visits, the CHW and the pregnant woman will jointly identify a room in the house or place outside the house that allows for counselling in private. Secondly, to reduce the risk of accidental disclosure of a woman’s HIV status, the procedures in the CHW intervention are the same for HIV-uninfected as for HIV-infected pregnant women. Thirdly, database access is restricted to a limited number of study staff for data entry and extraction. In addition, all staff involved in the study have been trained before initiation of the study to ensure that they are aware of the rules regarding confidentiality and data protection. All computers that are used for data entry as part of this study are protected by an individual password known only to the data entry clerk. In addition, the study database on each computer, where data from particular ANC and PMTCT clinics is stored, is protected by an individual password. At MDH, all computers and the study database are protected by passwords. Only individuals who are physically present at the MDH facility in Dar es Salaam can access the central server database, i.e., it is not possible to gain access from outside the facility. At any point in time, only 5-7 specifically authorized data entry staff at MDH have access to data in the central server database. No other study researchers have access to de-identified data in the central database.

### Illness of participants

When CHWs detect illness in a family member during their home-based visits, they will encourage referral to an appropriate health facility to the best of their ability.

### Sustainability and scalability

This trial will provide critical information on the scalability and sustainability of WHO Option A versus B and on a CHW intervention to improve ANC and PMTCT uptake and retention. It achieves this by integrating the interventions directly into the public-sector healthcare system rather than building a parallel structure for the delivery of the trial interventions, and by assessing feasibility, acceptability, and cost-effectiveness, all of which are crucial to sustainability and scalability.

### Dissemination of findings

The Tanzanian Ministry of Health and Social Welfare (MHSW) has been involved in the planning of this study from the start. In addition, three MHSW staff are members of the study team. This collaboration will ensure the dissemination of our findings to the MHSW on a continuous basis through both informal (such as personal meetings) and formal channels (such as study meetings and reports). In addition, the results of the study will be disseminated to the respective participating wards, antenatal clinics, and health workers through personal meetings and reports. Globally, the results of the study will be published in academic journals and presented at relevant conferences.

### Health systems implementation trial

This study can be categorized as a health systems implementation trial in contrast to other types of trials such as clinical trials. The study is a *health systems trial* for several reasons:

For one, the study is implemented through the public-sector health system in two of the three districts of Dar es Salaam. The trial has only become possible because the Tanzanian MHSW and the National Institute of Medical Research (NIMR) have supported it. The interventions themselves are implemented through the public-sector health systems structures and processes in Dar es Salaam, using only health worker cadres that already exist in the Tanzanian public-sector health system.

Option B has been introduced through educating existing health workers in all the facilities in the randomly selected wards in the two study districts about this PMTCT option. The trial team continues to engage on an ongoing basis with the health workers providing PMTCT and ANC in all four arms of the trial to ensure high fidelity in implementing the different intervention exposures.

In addition, the outcomes are assessed using the real-life public-sector health system’s data collection systems. The trial team supports the routine data collection including the extraction of a wide range of routinely collected data into an electronic database and data quality control. While a few additional variables are collected specifically for this trial, most of the primary and secondary outcomes of interest are collected through the routine data collection mechanisms in place in the Tanzanian public-sector health system, irrespective of this study (see Additional file3).

Finally, all pregnant women who seek ANC and PMTCT services in the public-sector health system in the Kinondoni and Ilala districts of Dar es Salaam are offered to participate in the study. Unlike in clinical trials, only a few selection criteria apply and the follow-up of participants regarding exposure and outcomes is carried out largely through the public-sector health system and linkage of individuals across different routinely collected data forms.

The study is an *implementation* trial because it assesses (1) the effectiveness of PMTCT Option A and B and the CHW intervention under the conditions of a real-life health system in SSA that has performed imperfectly in the past in ensuring universal PMTCT coverage of HIV-infected pregnant women; (2) how the effects of PMTCT Option B are modified by a CHW intervention; (3) outcomes other than effectiveness that are essential to inform health policy and practice, including cost-effectiveness, technical feasibility, and the social acceptability of Option B and the CHW intervention; and (4) the effects of Option B and the CHW intervention on implementation outcomes such as patient satisfaction, quality of care, and health worker job satisfaction. In addition, the trial attempts to affect PMTCT-related outcomes through a CHW intervention that focuses on ensuring early and comprehensive uptake of ANC (the initial health system function that needs to perform well in order to detect HIV-infected pregnant women and to link them successfully to PMTCT), and good retention in ANC and PMTCT through home visits of pregnant women to support continued ANC and PMTCT attendance throughout the course of pregnancy.

## Trial status

The study is funded by Comic Relief - UK and the Elton John AIDS Foundation. The National Institute of Medical Research in Tanzania approved the study in July 2012 and the institutional review board of the Harvard School of Public Health approved the trial of the CHW intervention in January 2013. Randomization, recruitment of project staff, CHWs and additional community outreach nurses, and training of healthcare workers has been completed. Enrollment in the trial commenced in January 2013. The trial will be stopped in May 2014. The trial is registered at ClinicalTrials.gov under the registration number EJF22802.

## Electronic supplementary material

Additional file 1: HIV/AIDS, ANC, birth attendance, and MTCT in Tanzania.(DOCX 19 KB)

Additional file 2: **The facility- and community-based trainings in the** ***Familia Salama*** **trial.**(DOCX 17 KB)

Additional file 3: List of data sources for the Familia Salama trial.(DOCX 18 KB)

Below are the links to the authors’ original submitted files for images.

Authors’ original file for figure 1

Authors’ original file for figure 2

Authors’ original file for figure 3

Authors’ original file for figure 4

Authors’ original file for figure 5

## Abbreviations

3TC: Lamivudine
ANC: Antenatal care
ARV: Antiretroviral drug
ART: Antiretroviral therapy
AZT: Zidovudine
CBHC: Community-based healthcare worker
CD4: Cluster of Differentiation 4
EFV: Efavirenz
HCW: Healthcare Worker
HIV: Human Immunodeficiency Virus
LPV/r: Lopinavir/Ritonavir
MDH: Management and Development for Health
MHSW: Ministry of Health and Social Welfare
MTCT: Mother-to-child HIV transmission
MTUHA: Tanzanian health management information system
NIMR: National Institute of Medical Research
NVP: Nevirapine
PCR: Polymerase Chain Reaction
PMTCT: Prevention of mother-to-child HIV transmission
RDT: Rapid Diagnostic Test
SSA: Sub-Saharan Africa
TDF: Tenofovir Disoproxil Fumarate
UNAIDS: Joint United Nations Programme on HIV/AIDS
WHO: World Health Organization.
