# Supplementary material for: Evaluation of a community health worker intervention and the World Health Organization’s Option B versus Option A to improve antenatal care and PMTCT outcomes in Dar es Salaam, Tanzania: study protocol for a cluster-randomized controlled health systems implementation trial
Source: Trials. 2014 Sep 15;15:359. doi: 10.1186/1745-6215-15-359 (PMC4247663; doi:10.1186/1745-6215-15-359)
Supplement: Supplementary file 1 — Additional file 1: HIV/AIDS, ANC, birth attendance, and MTCT in Tanzania.(DOCX 19 KB) [file 13063_2013_2319_MOESM1_ESM.docx]

**Additional file 1. HIV/AIDS, ANC, birth attendance, and MTCT in Tanzania**

*HIV prevalence:*

Adult HIV prevalence (15-49 years; 2012): 5% [1]

HIV prevalence among pregnant women (2009): 6% [47]

*ANC attendance:*

% of pregnant women who, for the most recent live birth (2005-2010) [30], had

- ≥1 ANC visit: 98%
- 1 ANC visit: 4%
- 2-3 ANC visits: 51%
- ≥4 ANC visits: 43%

Number of months pregnant at first ANC visit for the most recent live birth (2005-2010) [30]:

- <4: 15%
- 4-5: 50%
- 6-7: 31%
- ≥8: 3%

*Births attended by skilled health personnel (SHP)^1^* [30]*:*

% of births attended by SHP (2005-2010) –

- Overall: 51%
- In urban areas: 83%
- In rural areas: 42%

*HIV testing:*

% of pregnant women tested for HIV (2010): 86% [48]

% of infants born to HIV-positive women receiving a virological HIV-test within 2 months of birth (2012): 32% [1]

*ARV coverage:*

% of HIV-positive pregnant women who received ARVs for PMTCT^2^ (2012): 77% [1]

% of infants born to HIV-positive women who received ARVs for PMTCT (2012): 74% [25]

*MTCT:*

New HIV infections among children in 2012: 14,000 [1]

MTCT rate (2010): 25% [48]

^1^ Skilled health personnel includes doctors, clinical officers, assistant clinical officers, nurses/midwifes, and MCH aides.

^2^ Excludes women who only received single-dose NVP at the onset of labor.

Abbreviations: ANC = antenatal care; SHP = skilled health personnel; ARV = antiretroviral drug; PMTCT = prevention of mother-to-child HIV transmission; NVP = nevirapine; MTCT = mother-to-child HIV transmission, MCH = maternal and child health.

**References:**

1. UNAIDS: Global Report - UNAIDS Report on the Global AIDS Epidemic 2013. Geneva: UNAIDS; 2013. [http://www.unaids.org/en/media/unaids/contentassets/documents/epidemiology/2013/gr2013/UNAIDS_Global_Report_2013_en.pdf]

25. UNICEF: Towards an AIDS-Free Generation. Children and AIDS: Sixth Stocktaking Report, 2013. New York City: UNICEF; 2013. **[**http://www.childrenandaids.org/files/str6_full_report_29-11-2013.pdf]

30. National Bureau of Statistics: Tanzania Demographic and Health Survey 2010. Dar es Salaam: National Bureau of Statistics; 2010. [http://www.nbs.go.tz/takwimu/references/2010TDHS.pdf]

47. UNICEF: PMTCT Factsheet 2012. New York City: UNICEF; 2012. [http://www.unicef.org/aids/files/hiv_pmtctfactsheetTanzania.pdf]

48. UNAIDS: Global Report - UNAIDS Report on the Global AIDS Epidemic. New York City: UNICEF; 2010. [http://www.unaids.org/documents/20101123_GlobalReport_em.pdf]
