# Supplementary material for: Evaluation of a community health worker intervention and the World Health Organization’s Option B versus Option A to improve antenatal care and PMTCT outcomes in Dar es Salaam, Tanzania: study protocol for a cluster-randomized controlled health systems implementation trial
Source: Trials. 2014 Sep 15;15:359. doi: 10.1186/1745-6215-15-359 (PMC4247663; doi:10.1186/1745-6215-15-359)
Supplement: Supplementary file 2 — Additional file 2: The facility- and community-based trainings in the Familia Salama trial.(DOCX 17 KB) [file 13063_2013_2319_MOESM2_ESM.docx]

**Additional file 2. The facility- and community-based trainings in the Familia Salama trial**

*Facility-based trainings (delivered in all arms of the study):*

- Two 10-day basic PMTCT trainings for a total of 90 facility-based health care workers^1^
- Four refresher PMTCT trainings lasting between 3.5 and 6 days for a total of 500 facility-based health care workers^1^
- Two 6-day trainings for 30 clinical mentors^2^

*Trainings in the community health worker intervention (only delivered in wards randomized to the community health worker intervention):*

- A 5-day training for 72 community health workers and for 54 community outreach nurses^3^
- A 2-day training in the use of a monitoring and evaluation tool for the community health worker intervention for 54 community outreach nurses
- A 3-day training in the use of a monitoring and evaluation tool for the community health worker intervention for 213 community health workers
- A 3-day annual refresher training for 141 community health workers
- A 2-day annual refresher training for 54 community outreach nurses

^1^ The facility-based health care workers include doctors, nurses, nurse officers, and community outreach nurses.

^2^ The clinical mentors are nurses. See the section quality control for details on the study’s clinical mentorship program.

^3^ The remaining community health workers had already received the training.

Abbreviations: PMTCT = prevention of mother-to-child transmission
