# Supplementary material for: Evaluation of a community health worker intervention and the World Health Organization’s Option B versus Option A to improve antenatal care and PMTCT outcomes in Dar es Salaam, Tanzania: study protocol for a cluster-randomized controlled health systems implementation trial
Source: Trials. 2014 Sep 15;15:359. doi: 10.1186/1745-6215-15-359 (PMC4247663; doi:10.1186/1745-6215-15-359)
Supplement: Supplementary file 3 — Additional file 3: List of data sources for the Familia Salama trial.(DOCX 18 KB) [file 13063_2013_2319_MOESM3_ESM.docx]

**Additional file 3. List of data sources for the Familia Salama trial**

Clinical registers:

*Facility-based registers routinely used in Tanzania:*

1. MTUHA^1^ book 4 (the drug ledger)
2. MTUHA^1^ book 6 (the ANC register)
3. MTUHA^1^ book 7 (the pediatric register)
4. MTUHA^1^ book 12- (the labor and delivery register)
5. MTUHA^1^ book 13 (the postnatal care register)
6. ANC register^2^
7. Labor & delivery register^2^
8. PMTCT care register
9. PMTCT log book
10. Mother-Child follow-up register
11. PMTCT medicine register
12. Form A3 (records the monthly dispensation of ARVs by facility)
13. Form A6 (records the monthly consumption of HIV test and dried blood spot kits by facility)

*Client-held clinical records routinely used in Tanzania:*

1. Reproductive and Child Health Card #1
2. Reproductive and Child Health Card #4
3. HIV Care and Treatment Card #1
4. HIV Care and Treatment Card #2
5. Dried Blood Spot Card

*Registers added by the Familia Salama trial:*

1. Community Outreach Register (used by community health workers when visiting women at home)
2. ANC Participant Register (to track ANC attendance and to link the community outreach register to the facility-based ANC registers)
3. Antenatal Defaulter Tracking Form (to collect information on women who missed an appointment or who were referred by a community health worker but did not book an appointment)
4. Postnatal Mother and Child Attendance Register
5. Postnatal Defaulter Tracking Form (to collect information on women who missed a postnatal care appointment)

*Additional data sources for the cost-effectiveness evaluation (see below):*

- Publicly available data on the interventions’ costs
- Time-motion study

*Additional data sources for the evaluation of health workers’ performance and other job-related factors:*

- Household questionnaire to assess community health worker coverage and acceptability
- Questionnaire to health workers in all four arms of the intervention on health worker job satisfaction and related aspects
- Patient-exit interviews to evaluate patient satisfaction, quality of care perceptions, and related aspects

*Data sources for the population-based health survey (see below):*

- Household questionnaire

^1^ MTUHA is the acronym for Mfumo wa Taarifa za Uendeshaji wa Huduma za Afya, which is the Tanzanian health management information system.

^2^ The Tanzanian Ministry of Health & Social Welfare discontinued these registers in October 2012 (the variables collected in these registers were integrated into an updated version of the MTUHA books).

Abbreviations: ANC = antenatal care; PMTCT = prevention of mother-to-child HIV transmission; ARV = antiretroviral drug.
