# Supplementary material for: Evaluation of a community health worker intervention and the World Health Organization’s Option B versus Option A to improve antenatal care and PMTCT outcomes in Dar es Salaam, Tanzania: study protocol for a cluster-randomized controlled health systems implementation trial
Source: Trials. 2014 Sep 15;15:359. doi: 10.1186/1745-6215-15-359 (PMC4247663; doi:10.1186/1745-6215-15-359)
Supplement: Supplementary file 5 — Authors’ original file for figure 2 [file 13063_2013_2319_MOESM5_ESM.docx]

**Figure 2: The randomization scheme of the Familia Salama trial**

^
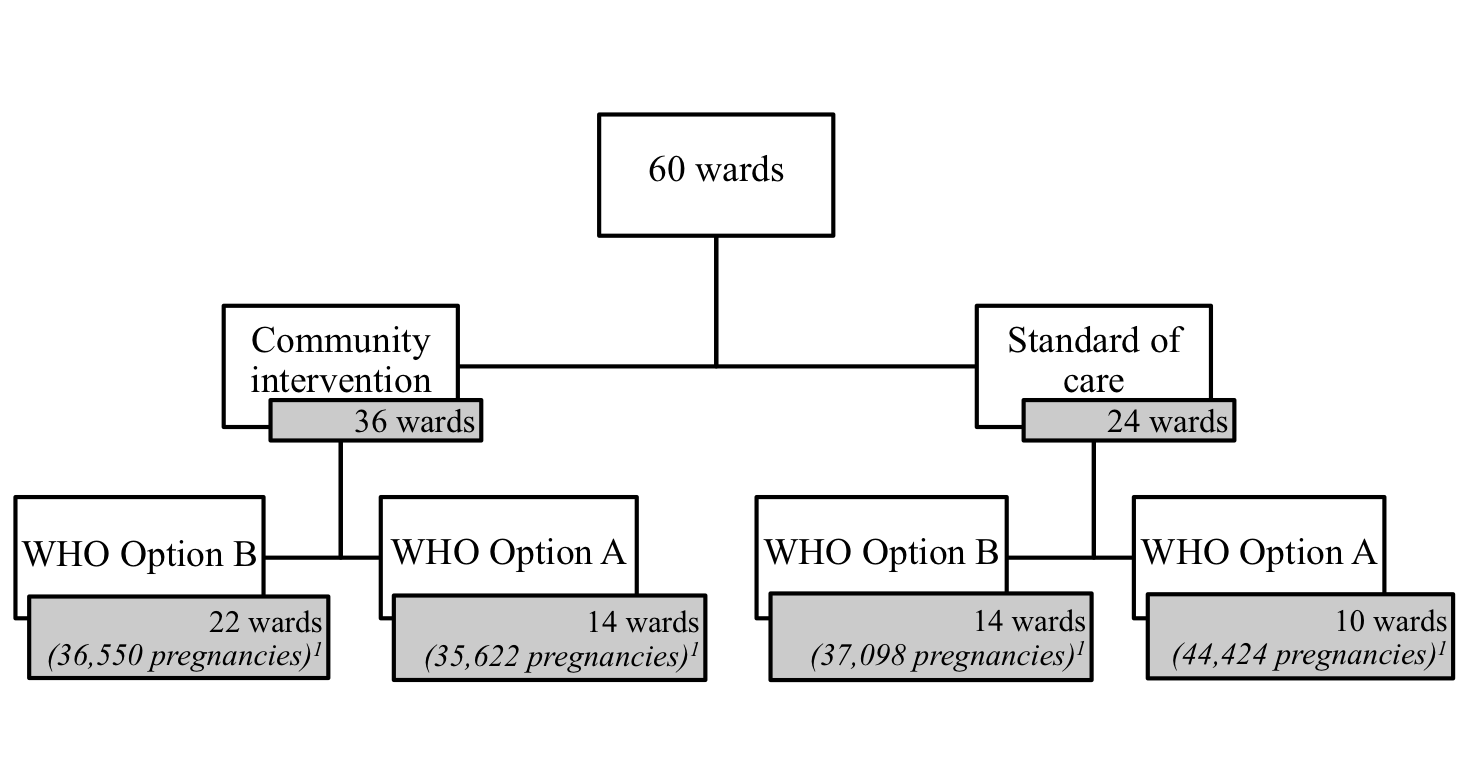
1^ = Projected number of pregnancies during the 17-month study period.
