# Supplementary material for: Evaluation of a community health worker intervention and the World Health Organization’s Option B versus Option A to improve antenatal care and PMTCT outcomes in Dar es Salaam, Tanzania: study protocol for a cluster-randomized controlled health systems implementation trial
Source: Trials. 2014 Sep 15;15:359. doi: 10.1186/1745-6215-15-359 (PMC4247663; doi:10.1186/1745-6215-15-359)
Supplement: Supplementary file 6 — Authors’ original file for figure 3 [file 13063_2013_2319_MOESM6_ESM.docx]

| **Figure 3. The facility- and community-based trainings in the Familia Salama trial**  *Facility-based trainings (delivered in all arms of the study):*   - Two 10-day basic PMTCT trainings for a total of 90 facility-based health care workers^1^ - Four refresher PMTCT trainings lasting between 3.5 and 6 days for a total of 500 facility-based health care workers^1^ - Two 6-day trainings for 30 clinical mentors^2^ (for details on the mentorship program, see p. 26)   *Trainings in the community health worker intervention (only delivered in wards randomized to the community health worker intervention):*   - A 5-day training for 72 community health workers and for 54 community outreach nurses^3^ - A 2-day training in the use of a monitoring and evaluation tool for the community health worker intervention for 54 community outreach nurses - A 3-day training in the use of a monitoring and evaluation tool for the community health worker intervention for 213 community health workers - A 3-day annual refresher training for 141 community health workers - A 2-day annual refresher training for 54 community outreach nurses   ^1^ The facility-based health care workers include doctors, nurses, nurse officers, and CBHCs.  ^2^ The clinical mentors are nurses. See the section quality control for details on the study’s clinical mentorship program.  ^3^ The remaining community health workers had already received the training.  Abbreviations: PMTCT = prevention of mother-to-child transmission; CBHC = community-based healthcare worker |
| --- |
