# Supplementary material for: Evaluation of a community health worker intervention and the World Health Organization’s Option B versus Option A to improve antenatal care and PMTCT outcomes in Dar es Salaam, Tanzania: study protocol for a cluster-randomized controlled health systems implementation trial
Source: Trials. 2014 Sep 15;15:359. doi: 10.1186/1745-6215-15-359 (PMC4247663; doi:10.1186/1745-6215-15-359)
Supplement: Supplementary file 7 — Authors’ original file for figure 4 [file 13063_2013_2319_MOESM7_ESM.docx]

| **Figure 4. The endpoints of the Familia Salama trial**  *Primary endpoints:*   - Proportion of pregnant women making at least four antenatal clinic visits (as recommended by the WHO) - Proportion of pregnant women delivering at a health care facility - Proportion of HIV-positive women receiving PMTCT - Proportion of HIV-exposed infants who received a confirmatory HIV test by 6 weeks after the cessation of breastfeeding - Proportion of infants born to HIV-infected mothers who have acquired HIV by 6 weeks after the complete cessation of breastfeeding   *Secondary endpoints:*   - Proportion of pregnant women who were tested for HIV during pregnancy or labor and delivery - Number of weeks of gestation at which pregnant women attend ANC for the first time - Proportion of HIV-infected pregnant women who completed PMTCT - Proportion of HIV-exposed infants who received PMTCT   *Additional endpoints:*  In addition to the designated primary and secondary endpoints, we assess the causal impact of the interventions on a range of other endpoints, which capture the following concepts:   - Cost-effectiveness - Social acceptability - Patient satisfaction - Health worker satisfaction |
| --- |
